# Diseases of the Liver as a Direct Cause of Intestinal Disturbances*Read before the California State Veterinary Medical Association.

**Published:** 1894-10

**Authors:** D. F. Fox

**Affiliations:** Sacramento, Cal.


### DISEASES OF THE LIVER AS A DIRECT CAUSE OF INTESTINAL DISTURBANCES*

By Dr. D. F. Fox.

This is a subject, gentlemen, which I have paid particular attention to in the last couple of years, and, owing to its insidious and complex nature, and it being a subject which is so difficult to ameliorate, and which, in my experience, does not readily yield to treatment, I naturally feel very anxious and ambitious to learn, from the exchange of ideas of those present here to-night, more about the pathology and treatment of intestinal disturbances. With this end in view, I have attempted to advance a few ideas, in which I hope to show that the majority of intestinal diseases are due to hepatic disturbance.

In order, therefore, to approach this subject in a comprehensive manner, we will first take up the consideration of the liver and its physiological properties.

As you all are aware, the liver being the largest as well as one of the most complex and important glands in the animal economy, and as it has some very essential and rather complex functions to perform, it is very easy to understand how any deviation from its normal condition will cause derangements of the alimentary tract.

The most important, and one of the chief, functions the liver has to perform, is the secretion of bile, the only one which directly pertains to the subject under consideration. There are, however, other functions, notably the one that controls the effect upon the blood during its transit through the hepatic circulation, whereby the blood is fitted for its subsequent purposes in the animal economy.

The secretion of bile is continually going on, but it appears to be greatly retarded during the period of fasting, and accelerated on the prehension of food, as has been shown by different experiments made by establishing a fistulous opening on the outside; it was noticed that during the period of fasting there was scarcely any bile discharged, but upon partaking of food an abundance of bile was discharged in a very few minutes.

The purpose served by the secretion of bile may be said to be

*Read before the California State Veterinary Medical Association.

of two principal kinds: excrementitious and digestive. From the peculiar manner in which the liver is supplied with much of the blood that flows through it, it is possible that this organ is excretory, not only for such hydro-carbonaceous matters as may need expulsion from any portion of the blood, but that it serves for the direct purification of the stream which, arriving by the portal vein, has gathered up various substances which may need to be expelled almost immediately after their absorption, for it is easily conceivable that many things may be taken up during digestion which are not only unfit for the purpose of nutrition, but which would be positively injurious if allowed to mingle with the general mass of blood.

The liver, therefore, may be supposed, placed in the only road by which such matters can pass unchanged into the general current, to jealously guard against further progress of effete matters, and turn them back again into an excretory channel.

One chief function of the secretion of bile is purification of the blood, by ultimate excretion of effete matter, yet there are many reasons for believing that it is in the intestines where it performs its most important function in the process of digestion. In nearly all animals the bile is discharged, not through an excretory duct communicating with the external surface, nor with a simple reservoir, as most secretions, but it is made to pass direct into the intestinal canal, where it mingles with the chyme directly after it leaves the stomach, an arrangement the constancy of which clearly indicates that the bile has some important relations with the food with which it is thus mixed. A similar indication of its digestive properties is furnished by the fact that the secretion of bile is most active, and the quantity discharged into the intestines much greater, during digestion than at any other time, although this activity may in part be due to the fact that a greater quantity of blood is sent to the liver through the portal vein at this time, and that this blood contains some of the materials of the food absorbed from the stomach and intestines which may need be excreted, either temporarily, to be afterwards reabsorbed, or permanently.

Bile is a somewhat viscid fluid, of a yellow or greenish color; it has a strong, bitter taste, and when fresh has scarcely any perceptible odor; it has a neutral and slightly alkaline reaction; its color and degree of consistency vary considerably, apparently independent of pathological changes in the liver. The saline, or inorganic, constituents of the bile are similar to those found in most of the other secreted fluids; the neutral phosphates, carbonates of sodium and potassium do not exist in the fluid state, but are formed during the act of incineration, and are found in the ashes. Oxide of iron is also said to be a common constituent of the ashes of bile; copper is usually found in healthy bile, and continually in biliary calculi.

Regarding the functions discharged by the bile in digestion, it may be said that it assists in emulcifying the fatty portions of the food, and thus rendering it capable of being absorbed by the lacteals. It has considerable antiseptic properties, and may prevent decomposition, putrefaction and fermentation of food during its passage through the intestines. It also acts as a natural laxative by promoting an increased secretion of the intestinal glands' and by stimulating the peristaltic action of the bowels, thereby assisting them in the propulsion of their contents.

Let us now for a moment consider in what manner we can arrive at a conclusion as to how we can account for the fact that intestinal disturbances are the result of morphological changes in the hepatic gland. As we have already stated, the liver is a very large organ, and has some very important functions to perform, of which, perhaps, the most important is the secretion of bile; bile, therefore, being a very essential substance in the process of digestion, it is perfectly plausible that an insufficiency or a preponderance of this important fluid will, and does, at once lead to intestinal derangements.

Being fully cognizant of the fact that one of the chief functions of bile is to act as an antiseptic, by preventing the food with which it comes in contact from undergoing the processes of decomposition and fermentation, we can readily see that an insufficiency of this very important material will allow the ingesta, or contents, of the intestines to become septic, putrid or fermentatious, a condition which is frequently demonstrated by the fetid aroma which always attends the fæcal matter of these cases. Again, bile being a natural carminative, an insufficiency of it will frequently be shown in the so-called cases of flatulent colic, which formerly were supposed to be due simply to fermentation of the food. Bile also acts as a natural purgative, by increasing the secretion of the intestinal glands; therefore, if the supply is curtailed, we are pretty sure to have indigestion, constipation, etc., follow as a sequel. If, however, the supply is increased beyond normal, we have as a sequel, diarrhœa, and I might state in this connection that it is my opinion that a great many cases of chronic diarrhœa are caused by a preponderance of bile being thrown into the intestines, a condition which is due to an over-active liver. Bile, acting as a local irritant, stimulates peristaltic action of the bowels, and increases their vermicular motion, thereby propelling or forcing the food through their channel; knowing this fact, we can readily see why a lack of this natural irritant which produces peristalsis will be followed by torpidity, paralysis, impaction, inflammation and gangrene of the intestines.

While it is possible to attribute, and perhaps trace, all lesions of the intestinal tract to be due to a morbid liver, yet I do not wish you to think that I advocate this idea, for I am fully aware of the fact that there may be, and are, numerous diseases of the intestines which have no connection with, and are independent of, any pathological changes in the liver, but as there are so many intestinal disorders which are liable to follow hepatic disturbances, I would suggest that a very rigid examination of the liver be made when called upon to administer to any animal affected with any disease of the visceral organs.

In establishing proof of, or in substantiating, the above statements, I will, with your kind permission, describe one of these particular cases, which, in my estimation, have their origin in the hepatic gland; for instance, you are called to see a case, which, on your first examination, will be lying down in the recumbent position, or standing in the corner of the stall, comparatively speaking, quiet, but upon closer examination you will notice that from time to time he will look around at his flank, or elevate the head and turn up the upper lip. If we now, supposing the animal to be lying down, make him get up, he will walk around the stall a few times, and will apparently make at intervals attempts to urinate, or to lie down again; finally, if he does not lie down, he will go into one corner of the stall, and stand there for some time in an attitude apparently of repose. Upon inquiring into the history of the case, you will be told that the animal, for the last two or three days, has had no appetite, either for solid or liquid matter. Upon still closer examination you will find the pulse running from 40 to 60, temperature 101 to 102; the conjunctiva will be of a dirty, reddish-yellow color. If you now manipulate the mouth, you will find that the tongue and mouth have a furred and soapy feeling, and the breath has a sour, fetid odor; upon auscultation of the flank, you will notice an absence of borborygmous sounds, that clearly indicate that there is an absence of peristaltic action of the bowels; upon percussion over the region of the liver on the right side, the animal usually evinces pain. The fæcal matter passed is dry, hard, and thickly covered with mucus, and is passed in small quantities; in some cases the sphincter anni has lost its contractibility, which leaves a permanent dilation of the rectum. If no relief is given, the animal will usually linger on in this condition, apparently with no perceptible change, for from eight to twelve days, when you will notice a change for the worse, when enteritis sets in, and the animal quickly succumbs to its baneful effects. If properly treated, however, a successful issue may be looked for in from three to five days.

Now, gentlemen, believing that all of you have, from time to time, been called to prescribe for like cases, when you felt, as I have done on numerous occasions, that you were, if you will permit me to use a hackneyed expression, "at sea" regarding the particular lesions you are to prescribe for, having this circumstance in mind, I concluded by presenting this matter before this honorable body, through whose experience and exchange of ideas I can reach a conclusion as to what manner I can therapeutically prescribe for these cases, so I can endeavor to remove the cause, and not, as I believe I have been doing heretofore, endeavoring to remove the effect.

SACRAMENTO, CAL.

